# A multiphoton microscope platform for imaging the mouse eye

**Published:** 2012-07-04

**Authors:** Omid Masihzadeh, Tim C. Lei, David A. Ammar, Malik Y. Kahook, Emily A. Gibson

**Affiliations:** 1 Department of Bioengineering, University of Colorado Anschutz Medical Campus, Aurora, CO; 2 Department of Electrical Engineering, University of Colorado Denver, Denver, CO; 3 Department of Ophthalmology, University of Colorado Hospital Eye Center, Aurora, CO

## Abstract

**Purpose:**

To demonstrate the ability of multiphoton microscopy to obtain full three-dimensional high-resolution images of the intact mouse eye anterior chamber without need for enucleation.

**Methods:**

A custom multiphoton microscope was constructed and optimized for deep tissue imaging. Simultaneous two-photon autofluorescence (2PAF) and second harmonic generation (SHG) imaging were performed. A mouse holder and stereotaxic platform were designed to access different parts of the eye for imaging. A reservoir for keeping the eye moist was used during imaging sessions.

**Results:**

Non-invasive multiphoton images deep inside the anterior chamber of the mouse eye were obtained without the need for enucleation. The iris, corneal epithelium and endothelium, trabecular meshwork region and conjunctiva were visualized by the 2PAF and SHG signals. Identification of the anatomy was achieved by the intrinsic properties of the native tissue without any exogenous labeling. Images as deep as 600 microns into the eye were clearly demonstrated. Full three-dimensional image reconstructions of the entire anterior chamber were performed and analyzed using custom software.

**Conclusions:**

Multiphoton imaging is a highly promising tool for ophthalmic research. We have demonstrated the ability to image the entire anterior chamber of the mouse eye in its native state. These results provide a foundation for future in vivo studies of the eye.

## Introduction

The anterior chamber of the mouse eye is a complex structure that closely resembles that of the human eye [1]. Many human ophthalmic diseases can be modeled and studied in the mouse eye including that of elevated intraocular pressure (IOP) and glaucoma [2]. The location of the conventional aqueous humor outflow system, important in the regulation of intraocular pressure (IOP) of the eye, between the cornea and the iris presents challenges for structural and functional testing which are aimed at better understanding the pathophysiology of glaucoma. Previous studies have shown that structural abnormalities in this region can lead to elevated IOP and development of glaucoma [3]. Therefore, early detection and clinical intervention in the prevention of this disease would greatly benefit from high resolution structural and functional imaging capabilities.

Current clinical techniques for imaging the eye include optical coherence tomography (OCT), confocal reflectance microscopy, ultrasound biomicroscopy and fluorescence imaging. In comparison with multiphoton microscopy, OCT has less spatial resolution, approximately 2–10 μm lateral and is not capable of achieving subcellular resolution. Confocal reflectance microscopy does allow subcellular level resolution, but is not capable of providing data on the functional status of imaged tissues. Ultrasound biomicroscopy is limited in resolution to ~25 μm and is also not capable of imaging subcellular structures or cellular function. For these reasons, multiphoton microscopy is a promising tool for ophthalmic imaging and could provide benefit in both research as well as clinical environments [4,5]. Specific imaging of the aqueous outflow system and trabecular meshwork (TM) has been performed with spectral domain OCT [6,7]. However, OCT is still not capable of obtaining functional information about the cells and collagen network in the outflow region and is limited in resolution. Three-dimensional micro-computed tomography (3D MicroCT) imaging with resolution on the order of a few microns has been performed on fixed human eye sections showing the outflow structures similar to histological imaging with light microscopy [8]. In contrast, multiphoton microscopy provides sub-cellular resolution and functional imaging capabilities using either intrinsic optical properties of molecules or optical markers such as fluorescent labels. We have previously demonstrated that multiphoton imaging can resolve the collagen structure of the TM without exogenous labeling as well as image epithelial cells in the TM by their intrinsic autofluorescence generated predominately by the cofactor nicotinamide adenine dinucleotide phosphate (NAD(P)H) [9]. Progression of primary open angle glaucoma (POAG) is indicated by a deterioration in the TM structure and in loss of epithelial cells in the TM, as found by electron microscopy studies [10,11]. A reduction in the dimensions of Schlemm’s canal in POAG has also been observed by histological studies [12]. Therefore, imaging this region of the eye may allow for an improved understanding of the pathophysiology of glaucoma, better diagnostic capabilities, and potential for assisting in new therapeutic drug discovery studies.

Multiphoton microscopy (MPM) is being increasingly used for ophthalmic research [9,13-25]. MPM provides sub-cellular resolution and considerable penetration depth as compared to confocal microscopy and is a powerful multi-modal tool for visualization of ocular structures by their inherent optical properties, without the need for labeling contrast agents [26,27]. Two-photon autofluorescence (2PAF) and second harmonic generation (SHG) are examples of different multiphoton imaging modalities. While both involve the simultaneous interaction of two infrared photons with molecules in the sample, they are fundamentally different phenomena and reveal different information about the molecular-level structure of the tissue [28-30]. 2PAF is generated by excitation of endogenous fluorophores such as the cofactors nicotinamide adenine dinucleotide phosphate (NAD(P)H) and flavin adenine dinucleotide (FAD) or melanin. SHG, on the other hand, is generated by non-centrosymmetric molecules or macro-molecular structures, such as collagen. Both SHG and 2PAF signals are arise from a two-photon process and therefore, the signal is produced selectively at the focus of the excitation laser where the intensity is greatest. This intrinsic axial sectioning property makes MPM ideal for three-dimensional imaging. In comparison with confocal microscopy, MPM can image at some depth in tissue because of reduced scattering of the infrared excitation light and greater detection efficiency of the emitted signal due to the elimination of the confocal pinhole and relay optics in the detection path. In addition, excitation with infrared light causes less thermal damage to the sample than visible wavelength excitation for the same intensities [31].

In previous results, we demonstrated the use of MPM to image the TM and nearby outflow structures within the intact, enucleated, unfixed mouse eye [15]. The mouse eye is ideal for these preliminary studies of MPM imaging due to its small size, thin sclera allowing for increased penetration depth of light for imaging and due to the well described anatomy of the outflow system in past research. The mouse eye anterior drainage system anatomy is very similar to that of the human eye and has been studied extensively, providing an excellent understanding of this model for future research in diseases of the eye. In the present study, we demonstrate MPM for in situ imaging of the mouse eye, to obtain the full three-dimensional images of the anterior chamber without any disruption to the tissue as occurs during the enucleation process. We describe our custom built multiphoton microscope specifically designed for ophthalmic imaging and the method for alignment of the mouse eye using a custom stereotaxic device. Our findings provide the foundation for achieving in vivo imaging of the mouse eye.

## Methods

### Animal protocol

The animal protocol for multiphoton imaging was approved by the University of Colorado Anschutz Medical Campus Institutional Animal Care and Use Committee (IACUC). The mouse strain C57BL/6 (Jackson Laboratory; Bar Harbor, ME) was selected for imaging because it has no documented ocular phenotype. C57BL/6 mice were obtained from retired breeder stock used in unrelated research projects. All mice were between 4 and 6 months of age. A total of 4 mouse eyes were imaged, all with qualitatively similar results. Representative images from one mouse eye are presented. For each experiment, the mouse was imaged immediately after euthanasia and each imaging session was around one hour in duration. Only one eye of each mouse was imaged during a given session after mounting and alignment of the eye with respect to the objective lens.

### Histology

After the multiphoton imaging experiments, the imaged mouse eye was enucleated and then preserved in 4% paraformaldehyde in PBS overnight at 4 °C. The eyes were embedded in paraffin and sectioned at a thickness of 6 µm. Tissue sections were stained with Mayer’s hematoxylin and eosin Y (H&E; Richard-Allan Scientific, Kalamazoo, MI). Bright-field imaging was performed using a Nikon Eclipse 80i microscope (Nikon, Melville, NY) equipped with a Nikon D5-Fi1 color camera and a Nikon 20X/0.30 Plan Fluor objective lens.

### Multiphoton microscopy

**Figure 1:**
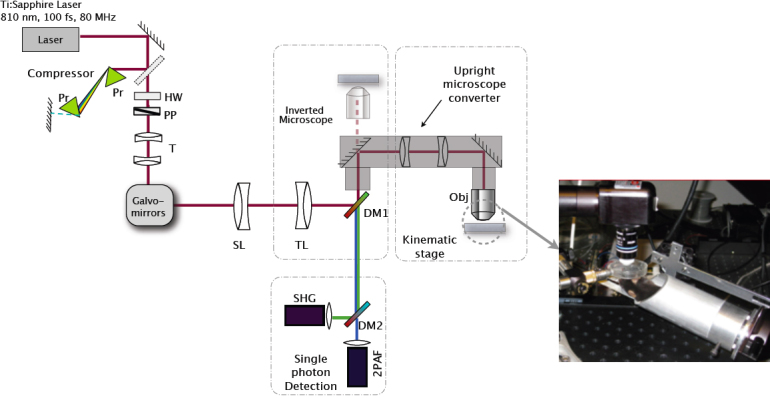
A schematic of the custom multiphoton microscope illustrating the beam path of the excitation laser and the emitted signals. The pulsed laser light is first sent through a prism compressor (to ensure the shortest pulse duration at the sample) and passed through two galvanometric scanning mirrors for raster scanning at the sample. The excitation laser beam travels through a scan lens (SL) and tube lens (TL) before being sent into the microscope through the side port. A custom made lens relay system is used to convert our inverted microscope (Olympus IX71) into an upright microscope for in situ imaging. The multiphoton signal from the sample is collected back through the microscope objective and separated from the excitation laser light with a dichroic mirror (DM1). An additional dichroic mirror (DM2) is used to separate the SHG and 2PAF signals, which are subsequently focused on separate photo-detectors. Abbreviations: prism (Pr), half-wave plate (HW), polarizer (PP), telescoping optics (T), objective lens (Obj). Inset shows a photograph of the custom stereotaxic device that holds the mouse and orients the eye position and angle with respect to the objective lens. A reservoir is held over the mouse eye to keep the eye moist during imaging sessions.

The images shown in this work were taken on a custom-built multiphoton microscope system. The system is built around an Olympus IX-71 (Olympus, Center Valley, PA) inverted microscope with several components that were custom designed and integrated for ophthalmic imaging capabilities (Figure 1). The excitation laser source is a pulsed infrared laser (Mai Tai HP; Spectra Physics, Santa Clara, CA) with a center wavelength of 810 nm emitting a train of pulses at an 80 MHz repetition rate. To optimize the multiphoton signal, the laser pulses are temporally compressed to the shortest pulse duration at the back aperture of the microscope objective by first passing through a prism compressor to compensate for the material dispersion due to the optical components in the beam path. Near transform-limited pulses of ~100 fs (the shortest pulse duration for the spectral bandwidth of our laser) were measured by a custom-built ultrafast pulse characterization apparatus based on the principle of frequency resolved optical gating [32]. The laser passes through a pair of telecentric lenses to increase the beam diameter to overfill the back aperture of the microscope objective to achieve a diffraction limited focal spot. Raster scanning of the laser focus across the sample is achieved by use of two non-resonant galvanometric mirrors (Cambridge Technology, Watertown, MA). A combination of scanning lens (SL) and tube lens (TL) is used to transform the angular translation of the laser beam at the scan mirrors into a lateral displacement of the laser focus in the focal plane of the objective lens. The pulse train with an average power of 15 mW (incident at the sample) is focused with an Olympus LUMPlanFL 40X/0.80 NA water immersion objective (Olympus) with a working distance of 3.3 mm.

The multiphoton signals generated by intense laser light interaction with the sample include second harmonic generation (SHG) and two-photon autofluorescence (2PAF). The emitted signals are collected in the epi-direction and separated from the excitation laser by a dichroic mirror (long pass at 685 nm, FF685-Di02; Semrock, Inc., Rochester, NY). Any residual excitation laser light is blocked by an additional sputter-coated, high-throughput shortpass 2-photon emission filter (ET700sp-2p ; Chroma Technology, Bellows Falls, VT). The SHG and 2PAF signals are spectrally separated with a dichroic mirror (T425lpxr; Chroma Technology) and bandpass filters (HQ400/20m-2p for SHG and HQ575/250m-2p 2PH; Chroma Technology) and detected by two separate high-sensitivity, photon counting detectors (H7422P-s40; Hamamatsu, Inc., Bridgewater, NJ). The signal from each detector is amplified (Model ACA-4–35-N, 35dB gain, 1.8GHz bandwidth, non-inverting amplifier; Becker and Hickl GmbH, Berlin, Germany) and converted to TTL (Transistor-transistor logic) pulses using a constant fraction discriminator (Model 6915; Phillips, Mahwah, NJ). The TTL pulses are sent to the counter inputs of a data acquisition card (PCIe-6259; National Instruments, Austin TX) with a maximum count rate of 80 MHz. Custom software was developed in Labview (National Instruments) running on a personal computer (Dell Inspiron, Round Rock, TX) to control the galvo-mirrors and read in the signal from the counters.

### Image analysis

Acquired multiphoton images were post-processed using Matlab (MathWorks, Natick, MA) and ImageJ software. Except when noted, all image dimensions are 256×256 pixels with a 13 μs pixel dwell time and frame averaging of two. The maximum image area is limited by the field-of-view of the objective lens and the scanning range of the galvanometric mirrors to ~140×140 μm^2^. Calibration of the image dimensions was performed by imaging a standard mesh with a calibrated grid size (Copper 400 Size Mesh; Ted Pella, Redding, CA).

To image over larger regions of the eye, tiling of many individual scans was performed. This was accomplished by translating the sample with a motorized stage (MS-2000; ASI, Eugene, OR) in the x and y directions with a step size of 140 µm to acquire a series of images. The images were then combined in software into one composite image (tiling). The effects of tiling can be seen in the reconstructed images as stripes with reduced intensity due to variations in the signal intensity across the field-of-view. This artifact can be eliminated by scanning a smaller region for each individual image, or by implementing more sophisticated image reconstruction software to correct for the intensity variations currently under development. A series of two-dimensional images was acquired at different depths and then projected in three-dimensions (3D) using ImageJ for 3D visualization. 3D animations were prepared using Zen 2009 software package (Carl Zeiss MicroImaging, Inc., Göttingen, Germany). In the 3D image reconstructions, the 2PAF and SHG signals are superimposed onto the same image for co-localized visualization. Appropriate noise reduction filtering (6-point smoothing function) and thresholding were applied to eliminate the noise from detector dark counts and to optimize visualization.

### Mouse holder

A custom stereotaxic device is used to position the mouse for multiphoton imaging of the eye. The holder is mounted on to the motorized stage that translates for tiling. The mouse holder incorporates two manual rotation stages to allow angular adjustments along two axes of rotation, to optimally position the mouse eye with respect to the objective lens. Acquisition of images at different depths is accomplished by translating the microscope objective vertically with a computer controlled stepper motor (MFC-2000; ASI, Eugene, OR) to adjust the focus knob on the Olympus IX-71 microscope platform. During the imaging experiments, it was important to keep the exterior surface of the eye moist. This was accomplished by a custom reservoir (Figure 1) positioned on top of the mouse eye to hold balanced salt solution (BSS) between the eye and the objective. The fluid in this reservoir also serves as the immersion liquid for the objective lens.

## Results

**Figure 2:**
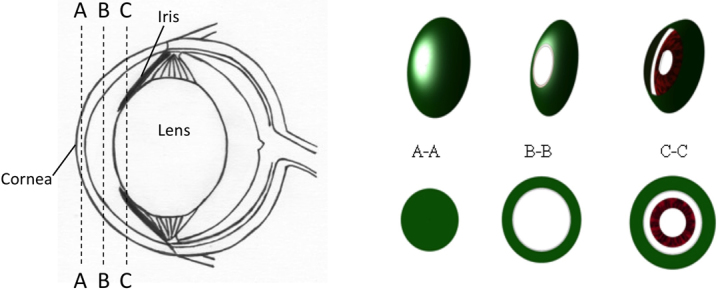
Diagram showing the different regions of the mouse eye that were measured with the multiphoton microscope, results shown in Figure 3. A-A represents a solid cut of the cornea from the top surface into the stroma. B-B is a cross-section of the anterior chamber filled with aqueous humor. Since the aqueous humor does not emit any signal, the sectioned image appears in the shape of a ring. C-C is a section deep inside the anterior chamber that includes the front of the iris.

The superior quadrant of the mouse eye was imaged with our custom multiphoton microscope from the surface of the cornea to the iris and anterior capsule of the lens. To visualize the different regions of the eye, we performed three-dimensional reconstructions of separate sections, illustrated schematically in Figure 2. Section A-A represents a solid cut through the top of the cornea where the image volume consists of the stroma and extends to the epithelium layer. Section B-B shows a solid cut through the cornea and into the anterior chamber. The ring appearance is noted due to the lack of signal generation from the aqueous humor located in the center of the volumetric section. Section C-C is the section deepest inside the anterior chamber capturing part of the iris, which generates a strong 2PAF signal (red) in the center while the outer ring consists of the cornea which predominately emits SHG (green).

**Figure 3:**
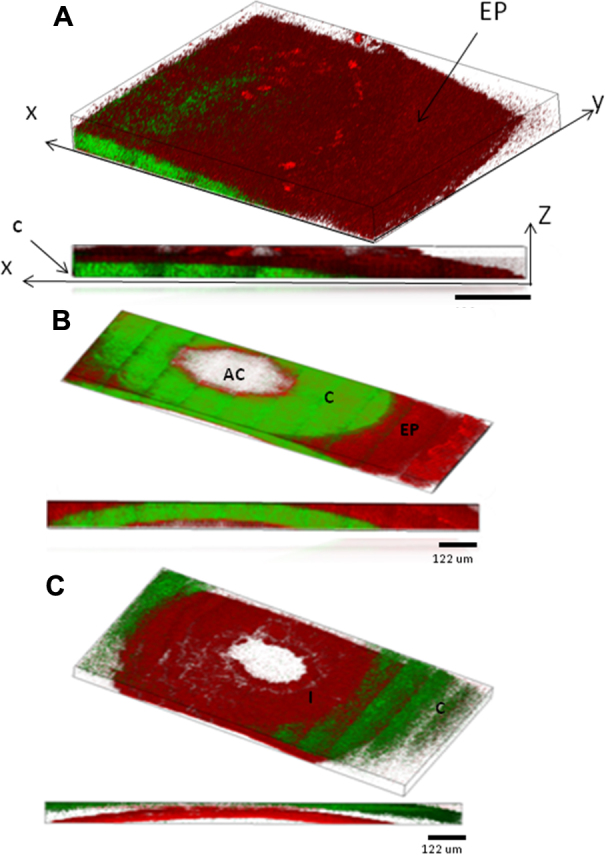
Three dimensional reconstructions of the images of the mouse eye taken with our multiphoton microscope. Below each 3-D reconstruction is a two-dimensional projection showing the side view of the image composite. The cornea (C) and iris (I) are indicated on the images. **A**: Image reconstruction of one quarter of the cornea section (indicated as section A-A in Figure 2). The epithelium layer (EP) is visible by the strong 2-photon autofluorescence signal (red; 2PAF) clearly delineating it from the stroma of the cornea that emits predominately second-harmonic generation from collagen (green;SHG). **B**: Image reconstruction of a cross section through the anterior chamber (AC; section B-B in Figure 2). The 2PAF signal on the outer rim of the image is from the epithelial layer on top of the stroma while the SHG signal is from the collagen in the stroma. **C**: Image reconstruction of the measured region deep into the anterior chamber that includes the iris (I) which emits 2PAF signal predominantly from the pigment granules.

**Figure 4:**
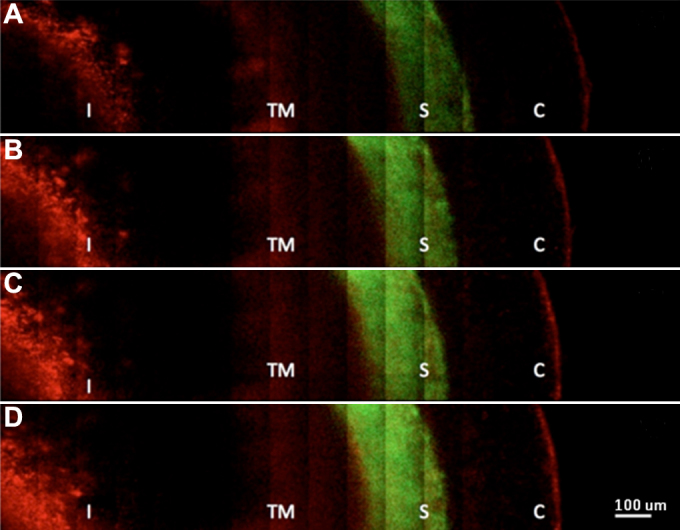
Two-dimensional tiled multiphoton images of the angle of the eye taken at different depths. Distinct features of the anatomy of the eye are visible. Images from top (**A**) to bottom (**D**) represent sections taken at different depths below the surface of the eye from (**A**) 580 μm, (**B**) 540 μm, (**C**) 520 μm, and (**D**) 500 μm. From left to right, we can see the iris (I), trabecular meshwork region (TM), sclera (S) and conjunctiva (C).

**Figure 5:**
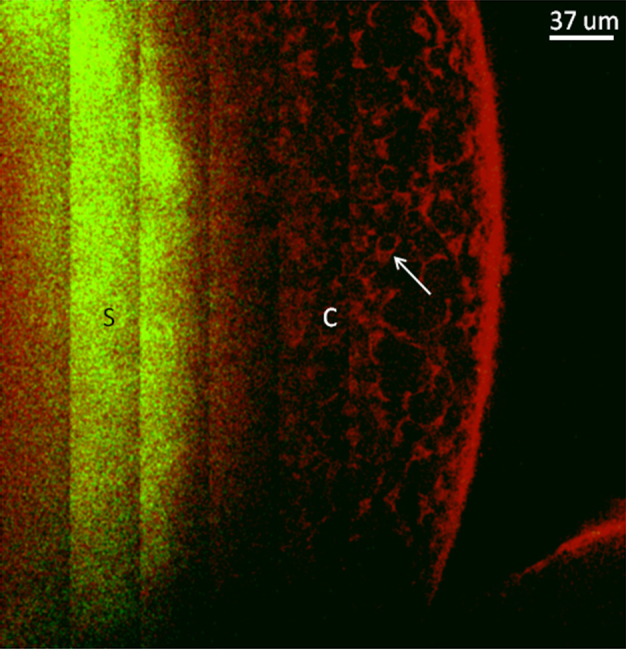
Two-dimensional tiled image showing a section through the sclera to the conjunctiva. The sclera (S) emits predominately SHG signal (green) while the conjunctiva (C) is visible by 2PAF (red). In this image, individual cells within the conjunctiva (see arrow) can be clearly resolved by their intrinsic autofluorescence from endogenous cofactors such as NAD(P)H and FAD.

Three dimensional image reconstructions of the data taken in the three sections of the anterior chamber of the eye are displayed in Figure 3. The images (Figure 3A-C) are shown starting from the exterior surface of the cornea and progressing deeper into the anterior chamber. False colors represent the SHG (green) and 2PAF (red) signals. Figure 3A shows the 2PAF signals from the epithelial cells at the surface of the cornea and SHG signal from the collagen-rich stroma further into the cornea, similar to our previously reported data in the enucleated eye [15]. The reconstructed three-dimensional volume is composed of 17 stacks in z (depth) of 4×5 tiled images (1 tile=136×136 μm^2^), each stack is separated in z by 10 μm. In Figure 3B, taken further into the eye, the signal is predominantly SHG (green) from the stroma of the cornea. The endothelium layer is distinct from the stroma by its strong 2PAF signal (inner red circle), while additionally in the same section, the epithelium layer is visible at the edge by its 2PAF signal (the outer red region). The reconstructed data consists of 12 z stacks (separated by 10 μm in depth) of 5×10 tiled images (1 tile=122×122 µm^2^). In section 3C, the iris becomes visible at a depth of 250 μm below the cornea. The nonlinear signal from the iris was exclusively 2PAF (red) from the pigment granules. The 3D image reconstruction consists of 16 z stacks (separated by 20 μm) of 12×6 tiled images (1tile=122×122 μm^2^). Figure 4 show a two-dimensional tiled view inside of the eye at different depths extending (from left to right) from the center of the pupil out to the outer edge of the iris and into the conjunctiva. The data are represented by 5×16 tiled images (1 tile=68×68 µm^2^). Four different depths are displayed in 20 μm steps starting at a depth of ~500 µm from the epithelium layer of the cornea. From left to right, one can identify the iris (I), trabecular meshwork region (TM), sclera (S) and conjunctiva (C). A higher resolution image of the conjunctiva is shown in Figure 5 where individual cell bodies are visible. The cells can be visualized by 2PAF predominately from endogenous fluorescent cofactors such as NAD(P)H and FAD [28].

**Figure 6:**
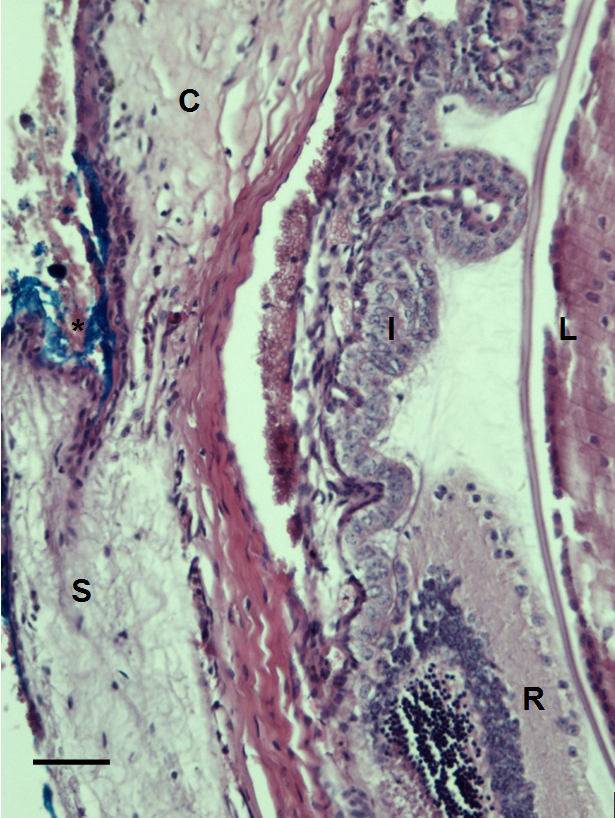
Histological section of C57BL/6 mouse eye imaged with our multiphoton microscope. Eye structures appear normal after imaging. No distortion or photocoagulation is noted in the tissues near the drainage angle of the eye. Blue dye was used to mark the orientation of the eye before enucleation (*). S=sclera. R=retina. I=iris. C=cornea. L=lens. The scale bar represents 50 µm.

Histology was performed to screen for potential damage to the tissue from the infrared excitation laser used in multiphoton imaging. After completion of the imaging experiment, blue dye was placed on the region of the mouse eye that was exposed to the laser light to identify the location for histology. Results from histology showed no visible damage to the tissue (Figure 6), indicating that we are able to image the eye structures at light intensities below the photodamage threshold.

## Discussion

In the current study, we demonstrate a multiphoton system capable of imaging deep inside the anterior chamber of a mouse eye without need for enucleation. Although imaging of the mouse cornea in vivo has recently been demonstrated [33], the current study is the first, to the best of our knowledge, in which multiphoton imaging was used to image the structures deep within the anterior chamber of a non-enucleated mouse eye. We have demonstrated the capability of imaging in the angle of the eye at depths of 600 μm and the ability to visualize the anatomy without the use of any external dyes or fluorescent labeling. Our multiphoton microscope is optimized for deep tissue imaging and is integrated with a custom stereotaxic device capable of rotation and translation with five degrees of freedom. The capability of both translational and rotational alignment is key to accessing different locations inside the anterior chamber. A fluid reservoir is designed to keep the eye moist for an extended period of time, necessary to obtain images of the tissue in its native state.

At our demonstrated penetration depth of 600 μm inside the eye, we can image a section through the entire anterior chamber, visualizing it in its native state. Due to light scattering and optical aberrations from inhomogeneities in the ocular tissue, the resolution of the microscope when imaging at-depth is reduced from the theoretical diffraction limited resolution of ~225 nm [34]. We are currently working on improvements to image resolution through several avenues including better aberration correction of the laser focus [35]. When imaging deep in tissue, care is taken to keep the excitation laser power as low as possible to avoid thermal damage. By using sensitive photon-counting detection, we were able to successfully image deep into the eye without any visible damage to the tissue, i.e., when repeating the scan over the same region, we did not observe any alteration in the imaged structures. Histology performed on the region of the eye that was imaged also showed no evidence of tissue damage. As an added safeguard for future in vivo studies, we can implement feedback controls to continuously monitor the multiphoton signals to detect any sudden increase that may indicate potential tissue damage, such as strong absorption by pigment-containing tissues. Upon detection of a rapid increase in signal, the laser power can be reduced during the scan to prevent any damage. From experiments at different laser excitation powers, our results indicated the pigmentation of the iris is the most susceptible to tissue damage most likely a result of the light absorbing properties of melanin. The cornea and sclera were more resilient to higher laser powers.

Currently, MPM imaging is much slower than OCT when imaging over the same volume. In spite of this disadvantage, for future clinical applications, MPM may have an advantage for high resolution “optical biopsy” of specific locations. MPM as an add-on to a standard OCT clinical instrument would potentially give both the fast scanning ability combined with high-resolution functional imaging in localized areas. Another limitation of MPM is the depth of penetration and decreased resolution at-depth due to scattering and tissue inhomogeneities. We are currently pursuing methods to address these limitations to higher resolutions in a way that will allow for practical use.

The study presented in this paper shows promising opportunities toward in vivo multiphoton microscopy for ocular imaging. The inherent sectioning capabilities and resolution of MPM along with its noninvasiveness are key to future clinical studies of live subjects and could add a wealth of information toward understanding diseases of the eye. For glaucoma, MPM imaging for studying the dynamic nature of TM cell survival in vivo has great potential. Recently, Tan et al. demonstrated that MPM imaging is capable of measuring the presence or lack thereof of TM cells on excised human corneal rims [36], however, in this study, they used exogenous markers and did not image through tissue to access the TM region. In our recent publication, we demonstrated use of coherent anti-Stokes Raman scattering (CARS) imaging for quantifying TM cells on excised human tissue without use of exogenous markers [16] and believe this is an additional multiphoton imaging method that could lead to clinical use in studying TM cells in live specimens when combined with our multiphoton imaging platform.
